# Book Reviews

**Published:** 1810-08

**Authors:** 


### CRITICAL ANALYSIS
OF THE
### RECENT PUBLICATIONS
IN THE
### DIFFERENT BRANCHES OF PHYSIC, SURGERY, AND MEDICAL PHILOSOPHY.

---

*An Essay on the Nature of Scrofula, with Evidence of its Origin from Disorder of the Digestive Organs; illustrated by a Number of Cases, successfully treated, and interspersed with Observations on the general Treatment of Children.* By RICHARD CARMICHAEL, Surgeon. 8vo. pp. 111.

IT has become of late so fashionable to attribute various diseases of the human body to a derangement of the functions of the *chylopoetic viscera,* that it excited no surprise in us to find an attempt made to refer scrofula to the same source; indeed, the functions of the digestive organs so readily become disordered, and their derangement so frequently *accompanies* most constitutional diseases, that that with our present imperfect knowledge of cause and effect, nothing is more easy than boldly to charge them as parents of the many untoward symptoms that denote any particular disease, while they only participate in common with other parts of the frame in numerous evils arising from some general cause. The greater the obscurity in which the nature and causes of any particular disease are involved, the more boldly may the assertion be made of that disease; scrofula therefore opens a wide field for speculation, and our author has availed himself of it with no little dexterity: it becomes us, however, to examine upon what grounds his hypothesis rests, and to bestow an attention to Mr. Carmichael's treatise, proportioned to the reputation he has already acquired, for we ought not to suffer doctrines to be promulgated by authority merely, which are not supported by facts and established by reasoning fairly deduced therefrom.

One of the most obvious requisites to a fair discussion of any subject is a clear comprehension of the terms employed; it becomes then an important question, what is the precise disease meant to be included under the name of scrofula? Here we find ourselves much at a loss for want of a definition by the author, for he shifts his ground so frequently, and takes so much for granted, which should have been proved, that we know not how to meet him. Assuming those symptoms to be scrofula which have never yet been allowed to be so, and which we should deny to be any part of that disease, and infering, that because these disputed symptoms appear together with certain other symptoms, they are caused by these latter, and that, as they all disappear under a certain treatment, *scrofula* is cured by that treatment, is a mode of reasoning neither fair nor correct, yet, upon this, and no better foundation, rest some of our author's peculiar doctrines in the treatise before us.

Among those who have chiefly contributed to hand down and propagate errors respecting this disease, the author would place in the foremost point of view Dr. Cullen.

"In support of this statement, it is only necessary to recollect his opinion that scrofula arises from "a peculiar acrimony of the fluids," that the disease 'rarely appears but in children whose parents had at some period of their lives been affected with it;' and his belief, that when it fails to appear in the children of scrofulous parents, it may discover itself afterwards in their offspring in the succeeding generations; as *also his reliance* upon mineral waters, and his supposition that they produce their beneficial effects by "washing out the lymphatic system."

We transcribe this passage to notice a mistake Mr. C. has fallen into, as to Dr. Cullen's *reliance* upon mineral waters, for the cure of scrofula, and we shall let the Doctor speak for himself on this subject. "For the cure of scrofula, we have not yet learned any practice that is certainly or even generally successful. The remedy which seems to be the most successful, and which our practitioners especially trust to, and employ, is the use of mineral waters; and and indeed, the washing out, by means of these, the lymphatic system, would seem to be a measure promising success; but in *very many* instances of the use of these waters, *I have not been well satisfied* that they had shortened the duration of the disease more than had often happened, when no such remedy had been employed."* Dr. Cullen states scrofula to be an hereditary disease, and Mr. Carmichael strongly objects to this opinion; but before we go further, let us see if Dr. Cullen and Mr. Carmichael really mean one and the same disease. We shall give the author's own description of the disease he has been so successful in treating, and his method of cure.

"In entering into a consideration of the circumstances which in children may produce disorders in the alimentary canal, it may be satisfactory to state a few cases, which prove that glandular swellings of the neck in infants are preceded and accompanied by a disordered state of the bowels; and that the removal of the former depends upon relieving the latter. Infants on the breast and in the second year are frequently affected with swellings of the glands at the upper and lower parts of the neck, and in the axilla, which make their appearance suddenly, and increase rapidly, often to a considerable size, until suppuration is established: when they break, or are punctured, they are difficult to heal, remain a long time open, and though at first they evince more of the phlegmonous than of the scrofulous inflammation, yet afterwards they often assume the *characteristic* appearances of scrofula. Having observed the connection between disorder of the primæ viæ and the symptoms of scrofula, I was induced to inquire into the state of the bowels in these cases; and I universally found that the evacuations of the child were either green, black, or slimy, for some time previous to the occurrence of the swelling. Within the last seven months I took notes of several of these cases which occurred at St. George's Dispensary, and in private practice; and I shall transcribe a few of them, as their history must convey a clearer idea of their nature than any general description.

"Case 1.—In the beginning of July, 1809, I was called upon to see an infant, three months old, affected with large tumours of a phlegmonous appearance on each side of the neck, immediately below the under jaw. On inquiry, I was informed that the child from her birth had never been regular in her bowels, and that her evacuations were constantly either of a green or black appearance. I directed a grain of calomel with six of rhubarb to be given every second night, and ten grains of prepared carbonate of lime, with two of carbonate of soda, every morning and evening.

"This course had soon a good effect. In four or five days the evacuations became natural in appearance, the tumours were poulticed

* First Lines, § 1753.

ticed until they broke, and in about a fortnight were perfectly healed.

"Case 2.—Edmond Burke, 14 months old, was brought to St. George's Dispensary on the 21st of August, 1809, on account of a tumour, hard, red, and painful, extending under the lower jaw from one ear to the other, the commencement of which was only observed by the mother four days before. His dejections, during the preceding six weeks, were of a green and sometimes of a black colour. I directed emmollient poultices to the tumour, a grain of calomel, with ten of rhubarb, to be taken every second night, and fifteen grains of carbonate of lime with five of soda, o rning and evening.

"Under this treatment, the evacuations soon exhibited a natural appearance, the tumour broke in a week, and after discharging a considerable quantity of matter, healed in a fortnight more, like any common abscess.

"I pointed out this case, as well as several others of the same description, to the attending physicians of St. George's Dispensary.

"Case 3.—William Jackson, 5 months old, was brought to me on the 21st of September, 1809, with an inflammatory tumour on the left side of the neck, which was only observed seven days before. His mother informed me that his bowels had not been regular si.ce his birth, but that he was attacked frequently by convulsions during the last fortnight, and that his dejections were of a green colour and sour smell. He was directed to take half a grain of calomel, with ten grains of prepared carbonate of lime, morning and evening. The frequent use of the tepid salt-water bath was also enjoined, together with warm cloathing and frequent hand rubbing.

"Under this treatment, in a fortnight, the evacuations became perfectly natural; about the same time the tumour broke, and soon afterwards healed without difficult .

"Case 4.—I was consulted about a child, 4 months old, on the 5th of October, 1809, who was affected with a large swelling, similar to those already mentioned, on the left side of the neck. She had been ordered poultices of sea-weed, by a physician of this city, I presume from an opinion of the scrofulous nature of the tumour.

"The mother requested advice at the same time for a disordered state of the infant's bowels, which she said had never been regular since her birth: under the plan of treatment mentioned in the last case, both complaints were removed in four weeks."

It certainly is no uncommon thing to meet with such phlegmonous tumours as the author describes, in young children, *accompanied with* a disordered state of the primæ viæ; we have seen numerous cases of them, and have for many years treated them nearly in the same manner Mr. Carmichael recommends, but we never never flattered ourselves that we had *cured scrofula* when these tumours suppurated and had healed; first, because we believed scrofula to be a disease that did not usually appear in the *first months* of the child's life; and next, because real scrofula is too unmanageable a disease to be cured in *two or three weeks*; scrofulous tumours are also much more indolent than those above described, which make their appearance suddenly, and increase rapidly, and as the author justly observes, "evince more of the phlegmonous than of the scrofulous inflammation;" they also appear at all seasons of the year, and in children apparently of very different temperaments, circumstances differing materially from the characteristic appearances of scrofula; but not to rest on our own authority, let us compare this affection with the disease named scrofula by Dr. Cullen, for to the accuracy of his history and description, we presume Mr. Carmichael will not object, whatever he may think of his opinions on the nature and causes of that disease.

"The scrofula generally appears at a particular period of life. It seldom appears in the *first* or *even in the second* year of a child's life; and most commonly it occurs from the second, or as some allege, and perhaps *more properly*, from the third to the seventh year. The scrofula generally shows itself at a particular season of the year; and at some time between the winter and summer solstice, but commonly long before the latter period. It is to be observed further, that the course of the disease is usually connected with the course of the seasons. Whilst the tumors and ulcerations peculiar to this disease, appear first in the spring, the ulcers are frequently healed up in the course of the succeeding summer, and do not break out again till the ensuing spring, to *follow again with the season, the same course as before.*" The tumors are without pain, and without any change in the colour of the skin; in this state, they often continue for a long time; *even for a year or two*, and sometimes longer: from the time they first appeared in the spring, they often continue in this way till the return of the same season in the next, or, perhaps, the second year after. About that time, however, or perhaps in the course of the season in which they first appear, the tumor becomes larger and more fixed; the skin upon it acquires a *purple*, seldom a clear redness, but growing redder by degrees, the tumour becomes softer, and allows the fluctuation of a liquid within to be perceived. All this process, however, takes place *with very little pain* attending it." *

We may fairly conclude, the *hard red painful* tumours Mr. Carmichael so easily and speedily cured, were not scrofula; this trifling circumstance, however, is no obstacle to our author, in constructing his hypothesis, which he attempts to establish by the following argument.

"But even supposing that these tumours were not in any way entitled

* First Lines, § 1740, 1742, 1744.

entitled to the name of scrofula, their connexion and dependance upon disorder of the alimentary canal, sufficiently establishes the point, *that swellings of the lymphatic glands may arise from, or be connected with, disorder of the digestive organs;* and this is all that is at present necessary for our purpose in tracing the symptoms of scrofula to their source."

We do not see how the admitting the connexion and dependance of those tumours upon disorders of the digestive organs, is *all* that is necessary to trace the symptoms of *scrofula* to their source, while we suppose these tumours *not in any way entitled to the name of scrofula.*

Resting then upon the solidity of the above quoted argument, the author proceeds, "Having satisfied ourselves that the first symptoms of scrofula are those which denote disorder of the chylopoietic viscera, and that disorder of those parts is in early life frequently followed by swelling of the lymphatic glands of the neck, let us now proceed to consider the several circumstances, which in childhood precede and induce this effect."

We must here observe, that *we* are not *satisfied* of the author's position; and lest our readers should be as little satisfied with it as ourselves, we will, by an extract from the volume before us, place the author's doctrine in a more connected point of view, and offer a few remarks, which naturally arise from the consideration of the proximate cause of scrofula being a disordered state of the chylopoietic organs.

"When it is considered that the first symptoms of scrofula are an enlarged belly, swelled upper lip, irritation at the nostrils, and irregularity of bowels, with green and black coloured fæces, all of which continue during the progress of the disease, and severally denote disorder of the digestive organs; and when it is also considered that disorder of those organs in children, is accompanied by the generation of an acid in the alimentary canal, we are justified in considering scrofula as a disease arising from, and generated by, disorder in the bowels, and that the treatment most likely to be successful, would be grounded on the indications of neutralizing the acid formed in the primæ viæ by alkalies and absorbent earths, of promoting a healthy secretion of the gastric juice, and of the bile, by exercise, tonics, and mercury, and of preventing, as much as possible, the further formation of acid in the bowels, by restricting the patient to a diet easy of digestion, and free from ascescency or disposition to the acetous fermentation."

So far from considering the symptoms above enumerated, as the pathognomonic ones of *scrofula*, we should conclude them to denote the presence of worms in the intestinal canal, or at least such a state of that canal as readily affords a nidus for those insects; while the healthy state of the digestive organs, during the existence of indurations of the glands and disease of the bones, the *acknowledged* symptoms of scrofula, would induce us not to think ourselves "justified in considering scrofula as a disease arising from, and generated nerated by, disorder in the bowels." At all events, the scrofula of Mr. Carmichael differs from the scrofula of other authors, especially from the disease so called by Dr. Cullen, as we have endeavoured to show; but there yet remains one point on which we must compare our author's opinion of scrofula, and Dr. Cullen's, and that is, its being, or not, an hereditary disease. The author thinks the popular notion of scrofula being hereditary, is founded in error, and he charges Dr. Cullen with propagating this error; he says, "in support of this statement, it is only necessary to recollect his opinion, that scrofula arises from a peculiar acrimony of the fluids;" that the disease rarely appears but in children whose parents had at some period of their lives been affected with it. In this quotation, *opinion* is confounded with fact; scrofula arising from a peculiar acrimony of the fluids was Dr. Cullen's *opinion*, its being hereditary was asserted as a matter of fact, and is only to be controverted by disproving that fact, by showing scrofula does not occur more *generally* in the children of scrofulous parents. Dr. Cullen's words are plain and unambiguous, and are capable of confirmation or refutation by an appeal to experience. "With respect to the influence of parents in producing this disease, it deserves to be remarked, that in a family of many children, when one of the parents has been affected with scrofula, and the other not; as it is usual for some of the children to be in constitution pretty exactly like the one parent, and others of them like the other, it commonly happens, that those children who most resemble the scrofulous parent, become affected with scrofula, while those resembling the other parent, entirely escape."* After all, it is a mere dispute of words; the author, while he admits advanced scrofula *to prevail* in particular families, denies the disordered state of the digestive organs, and the generation of acidities in the primæ viæ in children to be hereditary, and Dr. Cullen never asserted them to be so. The author calls that state of those organs scrofula, and Dr. Cullen never did. The following attempt to explain the fact of scrofula prevailing *in particular families*, is not satisfactory, nor does it notice the disease appearing exclusively in the children resembling the scrofulous parent.

"It must be acknowledged, however, that there are instances of the prevalence of this disease in particular families; but when this is the case, it is most probable, that instead of an hereditary taint, either a languid inert fibre predisposes to its attacks, or the same bad mode of rearing children (such as abandoning them to the negligence of hireling nurses) has been transmitted from one mother to another, and produces similar effects whenever it occurs."

With respect to the exciting causes of scrofula, much needs not to be said; while the author persists in calling weak digestion and acidities in the primæ viæ scrofula, he may fairly call all circumstances

* First Lines, § 1739.

stances which contribute to the production of these exciting causes scrofula, and they are too well known to require enumeration; one *fact* also renders the subject rather complex, scrofula being a disease of debility, and the persons in whom it is to occur in future life, being generally very weakly in infancy, digestion in them is imperfectly performed, and acidities, are produced; the scrofulous symptoms which afterwards appear, are then attributed by the author to this acidity, and weak digestion as its *proximate cause;* although during the existence of these scrofulous symptoms, neither weak digestion nor acidities can be discovered. How easily scrofula may be produced, and by what a variety of causes may well be imagined; and the author says, " in one of the worst cases of scrofula I have met with, and which continued even to manhood, the disease was attributed, by the mother of the patient, to his eating *a large quantity of manna* that was accidentally left in his way."

The treatment of scrofula will of course be founded upon the opinions of the author previously delivered, and a summary of it is contained in the following quotation:

" Having so far ascertained that indigestion is the proximate cause of scrofula, and that the remote causes *are weakness of fibre*, a want of due exercise, damp cold air, and acescent diet; the indications of cure are evidently to restore the digestive organs to the due exercise of their functions by the necessary attentions to diet, air, and exercise, and by the administration of medicines capable of correcting the morbific products of indigestion, and of exciting a healthy state of the digestive secretions."

The weakness of fibre which is here described as a *remote cause* of indigestion, has generally been considered as a predisposing cause of scrofula; whatever therefore will contribute to remove this weakness of fibre, will certainly be useful in the treatment of scrofula, and we approve of the exercise and other strengthening means recommended by the author, without assenting to the accuracy of his notion respecting indigestion being the proximate cause. The cases given in the former part of the book, exhibited the mode of treatment adopted by the author in the bowel complaints of very young children, and he has in addition given us several cases *of real scrofula*, treated in the same manner; and we shall now examine the result. We call it real scrofula, because in most of them the disease occurred at the usual age, viz. from five or six years to adult age, and the symptoms were such as have *hitherto* been denominated scrofula.

" I usually commence my plan of treatment by the exhibition of a purgative, in order to meet the symptoms which denote a foul and overloaded state of the primæ viæ; for this purpose I prefer calomel, because, while it empties the bowels, it induces an increased secretion of bile, by its *peculiar* effects upon the hepatic system. Two grains of calomel may be stated at the medium dose for children under twelve years of age, combined with ten of rhubarb. To adults I usually give three grains, and always exhibit the medicine at bed time, as it will be more likely to remain a longer interval in the bowels while the patient continues at rest, and is not exposed to any variation of temperature. Calomel given in this manner, increases the secretion of bile before we can suppose it to have entered the circulation, and through that channel affected the liver.

"In the morning following the exhibition of the calomel, a dose of vitriolated magnesia, proportioned to the age of the patient, generally produces four or five evacuations. For the first eight or ten days, I repeat the calomel every second night, and the neutral salt the following morning, which never fails to lessen the tumid state of the belly, and the swelling and itching of the nostrils and upper lip. The patient, instead of being reduced by these evacuations, is really improved in appearance, health and spirits.

"This plan does not interfere with the exhibition of the carbonates of soda and lime, which I give twice or three times daily to children under twelve years of age, the medium dose of which may be stated at half a drachm of the former to double that quantity of the latter. To those more advanced in life, I give these medicines in as large doses as the stomach can bear without inconvenience.

"After the first week or ten days, I generally omit the neutral salt entirely, and exhibit but one grain of calomel every second night, with ten grains of rhubarb; but in this I am determined by the degree of tumefaction of the belly, and swelling of the lip, invariably persisting in the neutral salt as long as these symptoms continue. Bitters or tonics I seldom employ, because their exhibition with the other remedies would be apt *to cloy or overload the stomach;* besides, I conceive that bitters can only be of service as a substitute to the bitter resinous principle of the bile, when that secretion is vitiated or deficient. And if the mercurial stimulus is sufficient to promote a healthy secretion of bile, the use of bitters would be superfluous; and I have had sufficient experience to satisfy me, that the remedies I have recommended are in general sufficient to answer every necessary purpose."

This mode was followed by the most beneficial consequences; in three or four months, the symptoms receded, and except in a few obstinate cases, the patients are reported *cured.* It must be observed, however, that this plan of treatment was begun in all of these cases, in *June* 1809. Well knowing that the symptoms of scrofula, spontaneously recede at the latter end of summer, to return again the following spring, we require a longer time than has yet elapsed to convince us these patients have been cured. Indeed, in one of them (Ward), the ulcer of the leg had broken out in the January following; this is the case which was produced by eating manna. Several of the cases here described, occurred in the girls at a parochial school, and the author attributes the disease in them to their being deprived of their accustomed exercise. "In *a short time* from the commencement of this sedentary life, scrofula la began to make its appearance, and afterwards affected near a third of their number." It certainly is against the generally received opinion that scrofula can be produced in so short a time, by such a cause, in habits where there does not exist hereditary predisposition; and this appears more unlikely still, if we admit indigestion and acidities in the primæ viæ to be the proximate cause, for then we should suppose causes applied *immediately* to the organs of digestion would be more speedily followed by their proper effects; yet in the boy who ate a great quantity of manna, thereby instantly disturbing the digestive organs, scrofula did not appear, the author informs us, till *the following year*.

Ever since the author has been *satisfied* that scrofula depends upon the deranged action of the chylopoietic viscera, he has not failed, in the cases that came under his care, to make inquiries concerning the state of these organs. As a proof that his *opinions* are not to be overturned by trifling circumstances, we give the following quotation.

"To my inquiry concerning the state of her bowels, I was informed that she was regular, but *this I am inclined to doubt;* for, from *the view we have taken* of the effects of exercise and pure air, in promoting the action of digestive organs, *I cannot think it probable* that digestion could have been performed with the same regularity in a young person, who was deprived of the exercise and purity of air, to which she had been accustomed from her infancy."

The impression that remains on our mind in closing the volume is, that the plan of treatment recommended by the author in infantile diseases, is proper and judicious, little differing from that generally adopted by medical practitioners; that its utility in the advanced stages of scrofula is at least ambiguous, further time being required to make a fair estimate of it; and that the hypothesis of scrofula depending solely upon the deranged functions of the chylopoietic viscera as its proximate cause, to the exclusion of hereditary predisposition, is not only unsupported by, but is directly contrary to the actual and acknowledged facts in the course of that disease.

---

### The Edinburgh Journal, No. 23.

Art. 1.—*Medical Report for Nottingham.* By James Clarke, M. D. Physician to the General Hospital, and to the Vaccine Institution.

Dr. Clarke has here presented us with another Medical Report, which, like the former ones, is a proof both of his indefatigable industry and attentive observation. After some remarks upon the revolution which medical opinions occasionally experience, and the desiderata to be had in view by writers of future accounts of the Walcheren Fever, he has given us a case of Diabetes, which, notwithstanding a great variety of treatment was adopted, at length proved fatal. The dissection showed, what might have been expected from the course of symptoms, organic disease of the

 bladder, bladder, schirrus of its neck, and a cartilaginous structure of the urethra. Bleeding appeared to afford at least considerable relief to this patient, and Dr. Clarke seems to regard this remedy with a favorable impression. But what we are particularly inclined to attend to in this article, is the new theory of the disease suggested by Dr. Clarke, which we shall give to our Readers in his own words.

"It has been already observed, that in most of the cases of this disease, the individuals have been previously subject to profuse sweating, which was suddenly checked by external cold, by drinking cold liquids, or by eating acid fruits, and speedily afterwards the diabetic affection appeared. Reflecting on this circumstance, it appears very rational to conclude, that the kidney became the receptacle of this suppressed fluid, and that the sweating, not simply the perspiration, was kept up from the kidney. This revulsion, as the ancients would call it, is admitted to occur from the kidney to other organs, and the converse must be equally correct. 'When the urine is not excreted, on account of some defect of the kidnies, ureters, or bladder, it has been exhaled into the skin, ventricles of the brain, or into the whole cellular fabric. The perspirable matter of Sanctorius, though so fluid, is sent off by the urinary passages, and by fear, or by medicines, through the excretory villi of the intestines.' (Haller's Physiology, 8th edit. p. 100.) The kidney will then have to throw off this sweating fluid in combination with the urine, which, from the stimulus given by this unnatural irritation, would be secreted in greater quantity; and to this stimulus the urinary organs cannot become habituated, as it is to be supposed it will be subject to the same variation as the cuticular discharge, sometimes being mild, at other times irritating; and on this principle, the constant irritation of the urinary passages, and the phymosis, may be explained. In consequence of this increased action, the circulation in the kidney will be quickened; a greater supply of blood will be required. Hence the stomach is called into inordinate action, and the demand for food proportionably increased; the appetite becomes craving; it is no sooner gratified than digestion hastily commences; chyme is formed, from which the chyle is quickly separated; but from the urgent demand of the kidney, it cannot be completely formed into blood, and in consequence, passes into the kidney with the blood, and there mixing with the urine, gives to it the saccharine quality; thus we may explain the bulimia, dyspepsia, and sweetness of urine. 'Hence the increased celerity of the blood so easily forces the red globules through these tubes, (uriniferous tubes) and, by morbid relaxation, they transmit the true fat and the chyle, and the salts of the meat and drink.' (Haller, p. 385.) Whilst this increased action is kept up in the kidney, every successive increase of debility in any other organ, will throw the more on the kidney. The stomach suffers first, being kept in constant action, and irritated by the gastric juice, which the constant stant sense of hunger calls into this cavity; and the more action required from this organ, the quicker must be the circulation thro' it; and if the supply of blood be not adequate, and the balance preponderate in favour of the kidney, the stomach must suffer doubly by the irritation of the gastric juice, and the want of support, which at last render it unable to digest its contents, and the patient dies from inanition."

It frequently happens, that the most obvious and insurmountable objections to an hypothesis escape the notice of its framer; and we are apt to think this has been the case with Dr. Clarke: we trust, therefore, he will excuse our pointing out a circumstance or two which make against his opinions, and of which we must see an explanation before we can become converts to this new theory; for however ingeniously it is supported by its author, it yet appears liable to some difficulties and doubts, which we should thank Dr. Clarke to answer. If the increased flow of urine, in the first instance, is merely a vicarious discharge; if nothing but the "sweating fluid" is sent to the kidneys, to be thrown off by them in combination with the urine, we see no possible source of mischief, nor can we allow that any *unnatural irritation* is thereby produced, the two fluids being so exactly similar, we may almost say identically the same; if also we suppose urine to be secreted in greater quantity, we should not expect the constant irritation of the urinary passages and phymosis to be referable to this circumstance, since we should apprehend these symptoms would rather take place from a greater concentration of that fluid than from its increased quantity and greater dilution.

Admitting the increased action of the kidnies to take place, we do not see why a greater supply of blood will be required for the system, so that the stomach must be called into inordinate action, and the desire for food proportionably increased, without time being allowed for its complete formation into blood. Were this true, the same effect must follow from any excessive discharge or waste of blood from the system; in weakness produced from hæmorrhages, for instance, a greater supply of blood will be required; and if the demand for it is too urgent to allow sufficient time for the complete conversion of the chyle into blood, the chyle must be hurried through the circulation into the kidnies, for by that source most fluid matters not converted into blood are discharged, and diabetes would be produced. In extensive local inflammations, of whatever description, there is increased action, and the circulation in the part is quickened, a greater supply of blood to the part is therefore required; yet we do not find the stomach called into inordinate action, nor the demand for food increased. The stomach being irritated by the gastric juice, *which the constant sense of hunger calls into the stomach*, is certainly a novel idea, but perhaps not a very just one. Wherein resides this sense of hunger? and how it is produced? The converse of the proposition may be

 true, true, and the constant sense of hunger be produced by the presence of the gastric juice, perhaps in a vitiated state from some disease of the secreting organs.

The treatment to be pursued in adopting this theory, is thus detailed.

"In the commencement of this disease, every thing should be done to turn the cuticular discharge into its proper course; this is to be effected by bleeding, the warm bath, and the administration of nauseating doses of tartarised antimony, ipecacuanha, and other medicines that possess the power of acting on the skin, without proving diuretic. Thus, the acetite and nitrate of potash would be bad medicines in this case. This treatment is to be assisted by a spare diet of animal and vegetable food. Having thus restored the natural secretion to the skin, nothing more remains than to moderate it, and to adopt the prophylactic measure, to prevent a recurrence of the same disease. But should this plan not succeed, and the disease baffles our means, it must be considered chronic, and now depending on a laxity or debility of the extreme vessels in the kidney; which opinion, the appearance of the kidney, and the state of the blood vessels, tend very much to confirm. It is then to be treated by astringents, tonics, blisters to the loins, the warm bath, and nourishing diet."

Next follows an interesting case of Hemicrania, which proved fatal. Upon dissection there was discovered in the posterior lobe of the cerebrum a hard tumour, which measured, after the contents had been evacuated, in length two inches, in breadth one inch and a half, and in depth one inch and a quarter; it was firmly attached to the tentorium.

Dr. Clarke speaks of the advantage of a clear diagnosis *between* tumour in the brain and hemicrania; we should rather say, in cases of hemicrania it would be very desirable to ascertain if it depends upon organic disease of the brain; for whether there is a tumour or not, the symptoms still constitute hemicrania, of which the cause may be various.

Two cases are then given, in which Dr. Clarke's treatment appears to have been very judicious, and was attended with success. They are denominated Cases of Water in the Brain; but some doubts may reasonably be entertained if water was *actually contained* within the brain, especially in the case first described, since the symptoms are equivocal, and not decidedly diagnostic of that affection. The same observation applies to the next and last case contained in this Report, A Case of Dropsy in the Pericardium.

In cases of such ambiguous nature as these, conjecture is often the utmost we can attain to, and our treatment must be founded rather upon experience of what has been found useful in similar cases, than deduced from our actual knowledge of the real nature of the disease. The paper is concluded with a few observations on the importance of accurate attention to the circumstances necessary to be observed in the gathering and preserving Digitalis, in which which we entirely coincide. Physicians are frequently disappointed in their expectation of the effects of this medicine, from want of its being dispensed in a sufficiently active state by those to whom this branch of the art is entrusted.

Art. 2. — *On Apparitions.* By John Alderson, M. D. Read to a Literary and Philosophical Society at Hull, January 1, 1805.

Dr. Alderson, while he allows the belief in ghosts, spectres, and apparitions to be well founded, has endeavoured to controvert the popular opinion of their existence depending upon supernatural agency, by bringing forward several instances where such appearances were to be accounted for by natural causes only, from some bodily disease in the persons by whom these spectres were seen. It is foreign to our purpose to enter into any metaphysical disquisitions; we may only observe, that in those cases he mentions, where a deceptio visus was produced by a temporary disorder of the animal functions, we will readily allow such appearances to have been fallacious, and the offspring of a distempered imagination. But this *negative evidence* is necessarily confined to those individuals mentioned. Dr. Alderson's explanation will go far to *diminish the actual number* of supernatural appearances; but the possibility of such an occurrence, or even the existence of it, cannot be completely overturned, unless he is prepared to disprove relations which have hitherto been considered as perfectly true and authentic. Have not some of these spectres, whether real or imaginary, occasionally uttered prophetic predictions, which have been afterwards realised? If we deny the reality of the spectre, we must allow the existence of a degree of prescience in the individual, which it is no less difficult to explain. Dr. A. admits it to be perfectly natural that Brutus should see a phantom; his imagination might be disturbed by "mental anxiety, inordinate ambition, or guilt;" but was it equally natural that, in this perturbed state, he should, at the same time, foresee the event of the battle of Philippi?

Art. 3 — *An Account of the Epidemic Dysentery which prevailed among the Dutch Troops at the Cape of Good Hope, in* 1804 *and* 1805; *with Remarks on the Use of Calomel in the Treatment of this Disease* By Dr. Henry Lichtenstein, of Helmstadt.

This paper contains a good description of the epidemic dysentery, as it appeared at the Cape of Good Hope. The treatment at first was very unsuccessful, founded upon the stimulant plan of the Brunonian system. Calomel was at length given to the patients, at first sparingly, and afterwards more freely. "It was constantly observed, that the crises of the disease were accelerated by the calomel." The sequelæ of the disease were principally suppuration of the liver, induration of that viscus, and chronic diarrhœa. The Author mentions it as a remarkable fact, that the Hottentots accustomed, customed to the climate, suffered much in the army, although better clothed, better fed, and more cleanly than in their agricultural occupations; while those who remained in the employment of the colonists, exposed to all the unwholesome weather, undergoing much fatigue, and being badly fed, were much less affected by the epidemic.

Art. 4. — *Case of Herpes Exedens Vermiculatus.* By A. B. Grenville, M. D. Surgeon to his Majesty's Sloop Arachne.

This at first was a very troublesome and obstinate disease. Dr. Grenville produced a similar ulcer in a distant part by inoculation with the discharge. On examining a drop or two of the pus by an excellent compound microscope, he discovered in that fluid "a swarm of insects, of various length and size, and differently agitated."

"The cause of this scabies being now plausibly discovered, the therapeutic indication readily occurred to my mind. The sores were washed with diluted nitrous acid, for three consecutive mornings, and cerate applied, to defend them from external irritation. On the first of November they were anointed with ung. hydrarg. with which the cure of both the *original* and *artificial* eruption was finally obtained, within the space of another week. No allowance of spirits or wine was granted to the patient during his illness; and though entirely local, I frequently administered sweet acidulated beverages, and a grain of opium at night to faciliate rest."

Art. 5. — *Reply to Mr. Goodlad's Observations on Mr. Barlow's Theory "On the Origin of Urinary Calculi."* By James Barlow, Surgeon.

In this Reply, Mr. Barlow endeavours to justify his former opinions, and to obviate some objections, and what he conceives to be misapprehensions on the part of Mr. Goodlad. The whole matter in dispute must be left to the parties themselves, who, most probably, as generally happens in controversies, will each retain his own opinion, unconvinced by the arguments of his opponent.

Art. 6. — *Convenient Method of constructing a Steam Bath, with an Account of its Effects in a Case of Gastritis.* By William Forbes, Member of the Royal College of Surgeons in London.

It is here proposed to convey steam by means of a tube communicating with a common tea kettle, and inserted into an aperture at the bottom of a slipper bath, in which the patient is to be placed; all the advantages of a warm bath may in this way be obtained, and more speedily than by heating sufficient water in which to immerse the body. Great relief was obtained by this bath in the case of Gastritis related, but the cure was effected by bleeding *ad deliquium* on the eighth day of the disease.

*The Inquirer*, No. 19.

After a fair and candid comparative view of the advantages and disadvantages, as to his means of improvement in the situation of a Naval a Naval Practitioner, the Inquirer concludes, that "if the list of naval surgeons be said to contain few illustrious names, the cause must be sought for somewhere else than in the nature of their situation." This is true so far as regards the opportunities they enjoy for increasing their medical knowledge, and correcting their theoretical opinions by accurate observations; but how few persons enter into the service, who have laid a sufficient foundation, whereon to erect a goodly superstructure, or who are endowed with that talent of observation so requisite for improving the science of medicine? A man of liberal education, and of real talent and abilities, finds nothing *inviting* in the situation of a sea surgeon; respectability of rank does not tempt him, and he is frequently subjected to treatment from persons of less cultivated minds, and less refined feelings, which at once mortifies and disgusts him. No prospect of future honours cheers his labours, and his sole motive of choice is generally a present maintenance. The service is also commonly entered into by youth, at an age when it requires no little degree of ardour and love of science to overcome the temptations to pleasure and indolence which exist around them. The following sentiments are so correct and judicious, that we cannot but repeat them, with a wish to impress them upon the mind of every one who is really solicitous for the improvement of medical science.

"In every large town there are professional men in extensive practice, who hurry from patient to patient, who write an old prescription because it is familiar to them, or a new one because it is fashionable, receive their fees, and think no more of their patient or his disease. That the public should suppose such men possessed of much experience is a natural mistake; but it is our duty to controvert the absurd idea, too prevalent among professional men themselves, that it is necessary to see very many patients to become an experienced practitioner, or that, in walking the wards of a crowded hospital, much practice is to be seen. On the contrary, a limited practice is most favourable for reflection and professional improvement. A multitude of patients only serves to distract the student, and to confound him; and the remarkable cases, which he may never again meet with, attract his whole attention, and lead him to neglect those common diseases, which, in future, it will be his chief employment to cure."

---

*A Letter in Reply to the Report of the Surgeons of the Vaccine Institution, Edinburgh; with an Appendix, containing a Variety of interesting Letters on the Subject of Vaccination, and including a Correspondence with Dr. Duncan, Dr. Lee, and Mr. Bryce.* By THOMAS BROWN, Surgeon, Musselburgh. Edin. 1809.

*A Correspondence with the Board of the National Vaccine Establishment.* By the same Author. London, 1810.

Mr. Brown's opposition to vaccination is sufficiently known by his former publication on the subject, and the present two indicate no abatement abatement of zeal in the cause. Zeal, however, sometimes oversteps discretion, and that appears to have been Mr. Brown's case, if we are to judge by his correspondence with Mr. Bryce, which is here given; wherein Mr. Brown endeavours to evade the HOME questions put to him by Mr. Bryce, thereby admitting that some of his assertions in his former book cannot be maintained. As to the FACTS contained in that book, we cannot refrain from giving an extract of Mr. Welsh's Answer to Mr. Brown's Letter, soliciting information, and Mr. Brown's SINGULAR Reply thereto.

"I cannot conclude my letter, without expressing my regret that your Queries were not addressed to the medical practitioners here, prior to the publication of your book; the result of all the information I have been able to obtain in this town and neighbourhood being *completely at variance with that stated by you to have been received from this place.* I remain, &c.

"*Haddington.* "JOHN WELSH."

To this Mr. Brown replies,

"Far from regretting that I did not communicate with the medical gentlemen at Haddington, before I mentioned in my book that such cases had occurred there, I have daily reason to congratulate myself on the opinion I had formed of the extensive and alarming effects of system on the human mind; for had I done so, and afterwards *had been regulated by the information I would certainly have received, undoubtedly no such opinions would have been promulgated*, and which I now find many very respectable characters, both in and out of the profession, consider as entitled to attention and respect."

We think it unnecessary to enter minutely into a consideration of the main question; we would rather leave the decision of it to fair and candid discussion and future experience. We do not expect Mr. Brown's books to make many converts; defective arguments seldom derive much real strength from warmth of language, and personal abuse generally fails to operate conviction on the mind of an opponent. If Mr. Brown is *perfectly satisfied* of the justness of his own opinions, and is only solicitous for the public welfare, we do not see why he should be so very angry that the favourers of vaccination do not enter the lists against him, and defend what he thinks an untenable doctrine; or why he should *complain*, when speaking of the National Vaccine Board, that he "perceives in their conduct a design to allow the *facts themselves* silently and gradually to produce the extinction of the practice," seeing the "extinction of the practice" is the sole object he professes to have in view.

---

*A Memoir on the Physiology of the Egg.* Read before the Linnean Society of London, by J. A. PARIS, M. B. Physician to the Westminster Hospital. London, 1810.

THIS interesting and philosophical Essay commences with a few cursory observations on the opinion that all animal and vegetable productions proceed from eggs or seeds.

"The

"The extensive range which the *Ovipari* form in the scale of animated existence, renders the organization and developement of the Egg, a subject of great interest to the naturalist, whilst the hope of ascending to the source of vitality, by contemplating life at a period when the number and complication of its functions are the fewest, becomes a powerful inducement to the physiologist, to pursue the investigation; hence we find, that the philosophers of every age and nation have devoted much time and labour to this inquiry; but unfortunately for the earlier promotion of science, the influence of chemical powers in the scheme of animal life has but lately been duly appreciated; many beneficial results, however, have already attended this discovery, and the most exhausted topics of natural history have assumed new and unexpected aspects. The author therefore of this memoir, may reasonably hope to escape the censure which must otherwise have awaited the adventurer who could presume to beat the field, which has before been so ably explored by the indefatigable and united labours of Fabricius *ab aqua-pendente*, Harvey, Malpighi, Spallanzani, Hunter, and others equally illustrious.

"A powerful phalanx of philosophers maintain, with much plausibility, that the *Egg** is the universal womb of nature, and that *oviparous* differ only from *viviparous* animals, by the latter breaking their bondage before they escape from the parent. Concerning the truth however of this opinion, which is comprehended in the popular aphorism, '*omnia ex ovo*;' or the success with which the eloquent Count de Buffon has levelled his shafts against the partisans of the ovular † system, I will leave to be decided by abler disputants. The observations which I beg to submit to your notice, do not involve either theory, but are connected only with those animals that are *oviparous*, in the common acceptation of the term—that is, *who deposit a germ to be developed by causes totally independent of parental influence* as such.

"Amongst the countless multitudes and varieties of animals, a very small proportion only produce living offspring; thus the immense tribes of birds, fishes, amphibious animals, and insects, with comparatively ‡ few exceptions, propagate their species by the intervention

---

* *Egg*, the word *ovum* seems to be derived from the Greek word *οιον*, *solitarium*, because (unlike most *uteri*) it produces but one offspring.

† The system of the Ovarists has been preferred by Harvey, Steno, Malpighi, Valisnieri, Dulamel, Nuck, Littre, Swammerdam, Haller, Spallanzani, Bonnet, &c.

‡ Some fish are viviparous, E. G. *Muræna Anguilla*, or Eel; *Blennius Viviparous*, &c. Amongst the *Amphibia*, we may notice the *viper*, which brings forth its young alive, and hence probably derives its name, "*quod vivum pariat*." Spallanzani considers also the production of frogs as rather of a viviparous than oviparous nature; this rudiment however of the future animal, certainly partakes as much of the nature of an egg as tervention of the egg; nor is this mode of generation either accidental or unimportant. Had the winged inhabitants of the air been *viviparous*, the burthen of gestation would have impeded the action of their wings, and so far increased their gravity, as to have rendered them incapable of the exertion of flight. The rigid and unpliant coverings of *crustaceous* animals, would oppose the expansion necessary for the growth of a fœtus; and it is evident, from the structure and habits of the tribe of serpents, that if they had been *viviparous*, their offspring must, from their tortuous flexions, and the friction arising from their progressive motion, have been constantly exposed to injury and destruction; and lastly, the *multiparous** nature of insects and fishes, at once convinces us how impossible it would be to engender them by any other mode than that which nature employs."

As the author confines himself in this memoir to the physiology of a particular description of eggs, he proceeds to lay down his distinctions and definitions of the several component parts of the eggs of birds, and the progress of their formation.

"The eggs of oviparous animals admit of an evident division into two classes, which I shall name. I. The Perfect, and II. The Imperfect. The former, which are deposited by the *Aves*, and some geneia of *Amphibia*, are completely covered by a hard shell, or membrane, and receive no additions after their exclusion; whilst the latter are deposited by most *Pisces*, and in general constitute a soft mass, not being protected by any external involucre. The observations contained in this memoir, relate more particularly to the egg of birds, their history however comprises whatever is interesting or important in the germs of inferior animals.

"In order that I may be better able to form a systematic relation of those new facts I wish to introduce to your notice, I shall briefly relate the successive operations by which the egg is formed in the body of the animal; the necessity of a more diffuse description,

---

of a fœtus, and may probably be considered as a connecting link between the two great classes. Insects likewise present us with exceptions, and several whimsical varieties; the *Aphides* for instance, lay eggs at the end of Autumn, when the young produced from them in the Spring, are viviparous during the whole Summer! the *Cocci* hatch their eggs before exclusion, and the young force their way through the abdomen of their mother; the *Onisci* carry their eggs in a particular receptacle, from which, in process of time, the young escape.

* Reaumur ascertained, that a single queen bee had laid, in the months of March and April, 12,000 eggs; and Lewenhoeck found that the *Musca carnaria* deposited 144 eggs, from which, in one month, were produced as many flies; so that supposing one half of these to be females, there would be, in the third month, 746,496 flies. The amazing fertility of fish may be illustrated by the *Gadus morhua*, or cod-fish, which will deposit among the rocks 9 or 10 millions of eggs; and again the perch, *Perca fluviatilis*, produces in April and May, 281,000 ova!

tion, is superseded by the minute and valuable details which may be found in the writings of Harvey and Malpighi.

"The rudiments of the *ovum* are first visible in the *ovarium*, which, in fact, is nothing but a congeries of *vitelli*,* or yolks, attached to the spine by a proper membrane; this repository is denominated, by Fabricius, the *vitellarium*, or *vitellorum racemus*, and may be considered as analogous to the *ovarium* of the *mammalia*, or to the *roe* of fishes. These *vitelli*, or *yolks*, generally vary in progression, from the size of a millet seed to that of an acorn; each according to its maturity, is successively detached from the rest, whence it descends a tube, called (from its resemblance to a funnel) "*infundibilum*," and arrives at the *uterus*, the internal surface of which is extended by spiral convolutions; here the albuminous fluids are secreted and transmitted to the egg, during its passage to the *fundus uteri* and *cloaca*, where it receives its external crust from the calcareous matter which is there deposited on it.

"The egg thus formed and completed, possesses every essential for its subsequent maturation, and requires only the emphatical agency of heat for its developement; this, in the different genera of animals, is conveyed by different media; in birds it is applied through incubation,† but in the *amphibia*, and other animals, the heat of whose bodies is inconsiderable and inefficient, the eggs are deposited in mud or sand, or exposed to the rays of the sun, by whose prolific influence myriads of beings are daily called into life and activity; or they are placed in other favourable situations, all of which are well known to the disciple of Linneus. It is however worthy of remark, that the medium through which heat is applied, is suitably varied in the same genus in different climates; thus, in Senegal, the ostrich abandons her eggs to be hatched by the burning sands, whilst, in the more temperate and congenial regions of the Cape of Good Hope, like other birds, she is inclined to incubation.

"The different species of *Æstrus* will afford us an illustration of the variety of situations in which the *ova* of animals are deposited; the *æstrus bovis*, or gad-fly, deposits them under the skin on the backs of oxen; the *æstrus hæmorhoidalis* in the rectum of horses; and the *æstrus ovis* in the frontal sinus of sheep, &c.; the excrescences also which are to be seen on cabbages, turnips, and other plants, always contain the egg of some insect. This faculty which animals possess of selecting an appropriate nidus for the reception of their ova, is truly astonishing, and affords a most beautiful illustration of the wisdom and intelligence which nature displays in the adaptation

* *Vitellus*, deriv. a *vita*, because it contains the embryon.

† There are also other animals which accelerate the evolution of their ova, by incubation; thus Bees in a hive, generate a considerable quantity of heat, without which their eggs would perish; and the *Testudo Mydas*, or common Turtle, deposits her eggs in the sand, and incubates during the night.

adaptation of means for the accomplishment of her ends: whether however the animal is directed in its choice by a moral or physical feeling, is not so evident; that is to say, whether it results from foresight, or from the blind obedience of a law of nature, which wisely connects the wants of the parent with the interests and welfare of the offspring; thus, for example, many animals, altho' termed *amphibious*, never resort to the water but to procreate their species; now are we to conclude that they repair thither because they know that this element alone can unfold their ova, or because, by so doing, they gratify some internal sensation, which renders their abode on dry ground unpleasant?"

A general account of the constituent parts of a *perfect* egg having been already given, this ingenious and diligent naturalist enters on the detail of its anatomy, if we may be allowed the expression.

"The parts of which the *egg* consists are, 1. *Vitellus*, or yolk, with its *capsule* and *cicatricula*; 2. *Albumina* with their proper membranes; 3. *Chalazæ*; 4. *Folliculus aeris*; 5. *Common membranes*; 6. *Exterior involucrum*, or shell; to each of which we will successively direct our attention.

"The *vitellus*, or yolk, is the part formed *in vitellario*, and is a yellow fluid contained in a membranous capsule, in which a white circular disk * is discernible; this is named *cicatricula*, and is the speck of entity, the germ that is to be developed into the animal. "In hujus gratiam," says Malpighi, "reliqua comproducta videntur." We here then have arrived to the earliest stage in which we can detect the animal's existence, our imperfect faculties will not allow us to ascend farther, and yet, even now, the body is formed, as the experiments and observations of Malpighi† most clearly demonstrate. The yolk is surrounded by a more tenacious fluid, to which the name of *albumen*, or commonly the white, has been assigned; this may easily be divided into two separate and distinct portions, each of which is contained in a concentric membrane; they differ from each other considerably in specific gravity, and seem to answer different purposes in the economy of the egg; the consistence of that which is exterior, is far less than the one which immediately envelops the yolk, and is consumed in the earlier periods of incubation,‡ whilst the internal and more viscid *albumen* seems reserved for the latter stages, when the chick must require a greater proportion of *generative matter* than at any other period of its development. Many of the ancient philosophers imagined that the chick was formed out of the yolk, and that the white afforded nutriment; such a theory must however be at once abandoned, when it is known that the *vitellus* suffers no change by incubation, except

* Fabricius supposes it to be a vestige of the ruptured *peduncle*, or that portion of membrane by which each yolk is connected to the vitellarium, and Æmilius Parisanus contends that it is the semen of the male.

† Vid. Marcell: Malphighi "de form: pull; in ovo."

designed to administer support, until its digestive organs can gain sufficient powers to perform their functions, and the beak a degree of firmness adequate to withstand the hardness of its natural food, "Ipsum animal," says Pliny, "ex albo liquore ovi corporatur, cibus ejus in luteo est;" doubtless however the *vitellus* answers also other purposes, or why should it be necessary to those birds * whose parents so sedulously supply them with nourishment?

"At each end of the egg, a white shining body is inserted into the capsule of the yolk, which extends into the *albumen*, in which it floats; these, from their resemblance in colour and transparency to *hail*, have gained the name of *chalazæ* or *grandines*, and, from having been formerly regarded as the sperm of the male bird, or *treddles*. Bellini supposes that they are composed of numerous canals, which open into the *amnios* or *cicatricula*, and send out their roots into the white, for the purpose of forming a communication between them; Munro however observes, that, "if they be canals, they cannot have the least communication with the cavity in which the chick resides, at any time, or in any state of the egg, otherwise than as they are both adhering to the membrane of the *vitellus*, upon which, or within which, no particular fibres, no canals, are stretched to the *cicatricula*." The *chalazæ*, says Harvey, appear to be the poles of the microcosm, and serve to connect the different parts of the egg, and to retain them in their due position; in addition to such an office, Denham ingeniously conjectures that as they divide the yolk into two distinct and unequal hemispheres, they will preserve the *cicatricula* (let the position of the egg be what it may), in the same situation; for since the *chalazæ* are specifically lighter than the white, the yolk is kept buoyant, and the *cicatricula*, as it resides in the smaller hemisphere, will be always uppermost; this, in my opinion, is the true theory of their use, for such a structure will not only preserve the *cicatricula* from the dangers of concussion, but by regulating its distance from the source of heat, it will ensure to it a more completely uniform temperature than could otherwise happen, and which is so essential to the evolution of the animal, that the smallest irregularity overthrows the nice balance of the different actions that are to mature it, and produces fatal effects. So solicitous therefore was nature to rescue the germ from the consequences of cold, that she has ordained other provisions, which seem equally as effective as the *chalazæ* for the preservation of its proper temperature; thus the *cicatricula* is on all sides surrounded by fluids, which are feeble conductors of caloric, these must therefore powerfully retard the escape of heat, and prevent the destructive chills which the occasional absence of the parent might otherwise induce. The eggs of

---

* Pigeons, for example, whose crops John Hunter ascertained to secrete a peculiar fluid during the breeding season, for the sustenance of their young.

many animals appear protected by an analogous apparatus; thus the ova of frogs, and other amphibia, are enveloped in spheres of mucilage, which the experiments of Spallanzani shew to be essential, as he found when this gluten was removed, the egg immediately perished. It is an evident fact that those fishes who retain their vitality, long after their removal from the water, as eel and tench, have the power of secreting a slimy fluid, with which they envelop their bodies; whilst, on the contrary, those which, when drawn on shore, quickly die, as for instance, mackrel, possess no such faculty, or at least only in a small degree; is it not therefore extremely probable that this albuminous matter, by repressing evaporation, and preventing (like the fluids of the egg) the escape of heat by its non-conducting nature, is the principal cause of this peculiar tenacity of life?*

"The hen bird seems herself conscious of the mischief that would accrue from an irregular or decreased temperature; she is often seen to make use of her bill to push to the outer part of the nest, those eggs that were nearest the middle, and to bring into the middle, such as lay nearest the sides. The Egyptians † however, by a nice adjustment of the temperature of their ovens, succeed in hatching a great proportion of the eggs subjected to artificial heat. The celebrated Reaumur introduced this method into France, and Sir James Hall has lately invented a regulating stove, by which an equable temperature for this purpose may be easily and advantageously procured. Sir Busick Harwood, the ingenious Professor of Anatomy at Cambridge, has frequently attempted to develop the egg, by the heat of his hot bed, but has succeeded only in producing monsters; the evident result of an unsteady application of heat. It must however be remarked, that deviations from the correct temperature, are injurious and fatal only in proportion to the degree of vital energy which the ovular embryon possesses; thus we learn, from the experiments of Spallanzani,‡ that the eggs of insects are better able to sustain the vicissitudes of temperature than those endued with more exalted vitality; thus it is, that the eggs of cold blooded animals bear with impunity such an increase or decrease of temperature, as is sufficient to destroy the animals themselves; for Spallanzani found

*tadpoles*

---

* Perhaps a prodigious accumulation of fat may also, sometimes, have a share in producing this effect; thus the *Silurus Glanis*, which is the fattest of all fresh water fishes, as it grows to the weight of 300 lb. lives very long after being taken out of the water.

† The inhabitants of the single village Berme, amongst whom only this art is practised, give life, by means of their ovens, to two-thirds of the eggs intrusted to their care, amounting in one season, which continues but for six months, to the astonishing sum total of 92,600,000. Corneille le Bruyn, tom. 2, has collected the observations of many travellers on this subject.

‡ Spallanzani. Tracts on the nature of animals and vegetables.

*tadpoles* and *frogs* to perish at 110°, bnt their eggs only at 133°. If we pursue this inquiry, and quitting the animal kingdom, descend into the scale of vegetable existence, where the energies of vitality are still more feeble and obscure, we shall find the same relative power of sustaining heat or cold between the plant and the seed, as we recognized between the animal and its egg.

( To be continued. )

